# Synthesis and Antimicrobial Screening of Novel 4-Substituted Phenyl-5-[1-(4-fluorophenyl)-1,3-dihydroisobenzofuran-5-yl]-2H-1,2,4-triazole-3-thiones

**DOI:** 10.1155/2014/439243

**Published:** 2014-11-20

**Authors:** Namratha Bhandari, Santosh L. Gaonkar

**Affiliations:** Department of Chemistry, Manipal Institute of Technology, Manipal University, Manipal 576 104, India

## Abstract

The paper describes a convenient method for the preparation of 4-substituted phenyl-5-[1-(4-fluorophenyl)-1,3-dihydroisobenzofuran-5-yl]-2H-1,2,4-triazole-3-thiones. The structures of the synthesized compounds are established by the results of LCMS, ^1^H NMR, ^13^C NMR, and IR and elemental analyses. The mercaptotriazoles are indicated to be in thione form by ^1^H NMR spectra. All the synthesized compounds have been screened for antibacterial and antifungal activities. Compounds **12d** and **12h** exhibit encouraging results, while the remaining compounds show moderate activities. On the basis of spectral studies, formation of 2-amino-1,3,4-thiadiazoles from the isobenzofuran acyl thiosemicarbazides **11(a–h)** is ruled out.

## 1. Introduction

**Figure 1:**
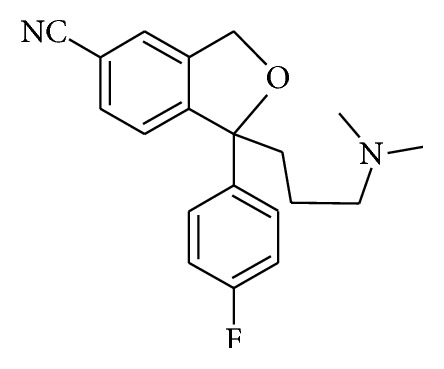
Structure of citalopram.

The benzo [c] furan system, trivially called isobenzofuran, has come to light from 1960s. There are records for the reactivity of substituted isobenzofurans as dienes in the Diels-Alder reaction [1, 2]. Nevertheless, its application for building natural and synthetic products has seen a setback. This is by reason of dearth of flexible methods for its synthesis. Citalopram (Figure 1), a well-known compound, affects serotonin metabolism in the brain and thus acts as an antidepressant drug [3]. Citalopram is prescribed for treating major depression [4], social anxiety [5, 6], panic disorder [7–9], diabetic neuropathy [10], premature ejaculation [11, 12], and poststroke pathological crying [13, 14]. 1-(4-Fluorophenyl)-1,3-dihydroisobenzofuran-5-carbonitrile is an intermediate in the synthesis of citalopram [15, 16].

The biological properties of 1,2,4-triazoles are well documented [17]. The pharmacological profiles of 3-mercapto-1,2,4-triazoles are tested meticulously for their antimicrobial [18, 19], anticonvulsant [20], and antidepressant activities [21]. 1,2,4-Triazoles from 1-(4-fluorophenyl)-1,3-dihydroisobenzofuran-5-carbonitrile is a venture to blend properties of two distinct heterocyclic entities.

An attempt to prepare 2-amino-5-substituted-1,3,4-thiadiazoles from substituted acyl thiosemicarbazides** 11** has failed to furnish the corresponding products. The decomposition of the starting material is indicated by ^1^H NMR and ^13^C NMR spectra. This reaction in acidic medium is dependent on both pH of the medium and substituents of acylthiosemicarbazides. The probable reason for the failure may be the steric hindrance posed by bulky isobenzofuran component for cyclization pathway.

This paper portrays the synthetic tactic for the preparation of 3-mercapto-1,2,4-triazole derivatives bearing isobenzofuran moiety and an assessment of their antimicrobial action.

## 2. Materials and Methods

### 2.1. Chemistry

The melting points are determined on Thomas Hoover apparatus and are uncorrected. The IR (KBr) spectra are recorded on Shimadzu 8300 Fourier transform infrared spectrometer. ^1^H NMR is recorded on Bruker AM 400 MHz spectrometer and ^13^C NMR is recorded on Bruker AM 100 MHz spectrometer using DMSO as solvent and TMS as an internal standard (Chemical shift in ppm). Mass spectral (MS) analysis is carried out on Agilent 6520 ESIQTOF MS with Ionization source ESIQTOF and acetonitrile as solvent (110 Volts). Elemental analyses are obtained on a Vario-EL instrument. Thin layer chromatography is conducted on 0.25 mm silica gel plates (60F_254_, Merck). Visualization is made with ultraviolet light. All extracted solvents are dried over Na_2_SO_4_ and evaporated with a BUCHI rotary evaporator. Reagents are obtained commercially and used as received.

#### 2.1.1. Procedure for the Synthesis of 5-Amino-3H-isobenzofuran-1-one (3)

To a cooled solution of SnCl_2_ (1.0 g, 5.27 mmol) in Conc. HCl (20 mL) add 4-nitrophthalimide** 1** (5.0 g, 0.026 mol) slowly under stirring. After complete addition, the reaction mass is stirred for 3 h at r.t. The reaction mass is cooled and the solid formed is filtered and washed with chilled water. The wet solid** 2** (4 g, 0.024 mol) is dissolved in 30% aq. NaOH solution (20 mL). Zn dust (2 g, 0.030 mol) is added to the mixture and refluxed for 4 h. The residue is filtered and the filtrate is acidified with Conc. HCl (20 mL). This mass is refluxed again for 2 h. Thereafter, the reaction mass is cooled and the pH is adjusted to neutral using liq. NH_3_. The solid formed is filtered and recrystallised from boiling methanol to afford 5-amino-3H-isobenzofuran-1-one** 3** as off white solid, yielding 3.0 g (83%), m.p. 186–188°C; IR (KBr pellet, cm^−1^): *ν* 3133 (NH), 3085 (aromatic –CH), 1644 (C=O), 1620, 1471 (aromatic C=C), 1044 (COC), 1022 (CN); ^1^H NMR (DMSO, 400 MHz): *δ* 7.55 (d, *J* = 8.0 Hz, 1H, ArH), 6.62–6.69 (m, 2H, ArH), 5.32 (s, 2H, CH_2_), 5.13 (bs, 2H, NH_2_); ^13^C NMR (DMSO, 400 MHz): *δ* 169.0, 141.7, 137.6, 133.0, 131.9, 128.9, 124.4, 70.5; Anal. Calcd. for C_8_H_7_NO_2_: C, 64.42; H, 4.73; N, 9.39. Found: C, 64.49; H, 4.69; N, 9.34.

#### 2.1.2. Procedure for the Synthesis of 1-Oxo-1,3-dihydro-isobenzofuran-5-carbonitrile (4)

Dissolve 5-amino-3H-isobenzofuran-1-one** 3** (2.00 g, 0.013 mol) in Conc. HCl (10 mL). Dilute with water (30 mL) and stir to make a homogeneous solution. Cool in ice and add NaNO_2_ (1.10 g, 0.016 mol) solution in water (2.5 mL) under stirring. To this cool diazonium salt solution add CuCN (1.42 g, 0.015 mol) slowly in small quantities with warming on a water bath at 60°C. Reflux for 30 min and then cool the reaction mass to get the title compound** 4** which is recrystallised from ethyl acetate, yielding 1.68 g (84%), m.p. 202–204°C; IR (KBr pellet, cm^−1^): *ν* 3043 (aromatic –CH), 2251 (CN), 1685 (C=O), 1670, 1444 (C=C), 1037 (COC); ^1^H NMR (DMSO, 400 MHz): *δ* 8.01 (d, *J* = 7.5 Hz, 1H, ArH), 7.65 (d, *J* = 7.5 Hz, 1H, ArH), 7.62 (s, 1H, ArH), 5.38 (s, 2H, CH_2_); ^13^C NMR (DMSO, 400 MHz): *δ* 171.1, 140.6, 137.8, 132.5, 131.7, 128.4, 125.7, 119.6, 69.4; Anal. Calcd. for C_8_H_4_NO_2_: C, 67.92; H, 3.17; N, 8.80. Found: C, 67.91; H, 3.19; N, 8.88.

#### 2.1.3. Procedure for the Synthesis of 1-(4-Fluorophenyl)-1,3-dihydroisobenzofuran-5-carbonitrile (7)

Under nitrogen atmosphere* p*-fluoro phenyl magnesium bromide (7.5 g, 37.73 mmol) is added dropwise to a stirred solution of 1-oxo-1,3-dihydro-isobenzofuran-5-carbonitrile** 4** (5 g, 31.44 mmol) in THF (50 mL) at 0–5°C. The mixture is stirred for 1 h at r.t. Add saturated NH_4_Cl to facilitate decomposition of magnesium complex. Put in some more THF and wash with water. Remove off THF and pour residue** 5** into methanol (10 mL). NaBH_4_ (0.58 g, 15.72 mmol) is added slowly for 30 min to the reaction mass and stirred for 2 h at r.t. Solvents are removed to get diol** 6** which is refluxed with 2N HCl (50 mL) for 2 h. The reaction mass is cooled and diluted with water. The product is extracted into methylene chloride, washed with water, and dried (anhy. Na_2_SO_4_). The solvent is removed and the residue is crystallized in IPA to provide 1-(4-fluorophenyl)-1,3-dihydroisobenzofuran-5-carbonitrile** 7** as pale yellow solid yielding 5.9 g (79%), m.p. 97-98°C; IR (KBr pellet, cm^−1^): *ν* 3013(aromatic –CH), 2246 (CN), 1699, 1479 (C=C), 1038 (COC); ^1^H NMR (DMSO, 400 MHz): *δ* 7.65 (s, 1H, ArH), 7.58 (d, *J* = 8.5 Hz, 1H, ArH), 7.31 (dd, *J* = 14.0 Hz, 2.6 Hz, 2H, ArH), 7.15 (d, *J* = 8.5 Hz, 1H, ArH), 7.09 (t, *J* = 7.6 Hz, 2H, ArH), 6.11 (s, 1H, CH), 5.29 (d, *J* = 2.0 Hz, 1H, CH), 5.18 (d, *J* = 2.0 Hz, 1H, CH); ^13^C NMR (DMSO, 400 MHz): *δ* 144.8, 140.5, 137.2, 133.3, 132.9, 131.3, 128.9, 128.4, 125.7, 119.6, 116.0, 84.2, 71.8; Anal. Calcd. for C_15_H_10_FNO: C, 81.08; H, 4.58; N, 6.36. Found: C, 81.10; H, 4.55; N, 6.39.

#### 2.1.4. Procedure for the Synthesis of 1-(4-Fluorophenyl)-1,3-dihydroisobenzofuran-5-carboxylic Acid (8)

1-(4-Fluorophenyl)-1,3-dihydroisobenzofuran-5-carbonitrile** 7** (5 g, 20.92 mmol) is added to methanol (50 mL) and 1N NaOH (20 mL). The mixture is refluxed for 3 hr. Completion of reaction is marked by TLC with mobile phase toluene : ethyl acetate = 7.5 : 2.5. After completion of reaction, the mass is cooled and acidified with 1N HCl. The solid formed is filtered, washed with water, and recrystalized from ethanol to give** 8** as crystalline white solid with a yield of 4.7 g (87%), m.p. 188–190°C; IR (KBr pellet, cm^−1^): *ν* 3012 (acid CH), 2912 (acid OH), 1707 (C=O), 1682, 1492 (C=C), 1039 (COC); ^1^H NMR (DMSO, 400 MHz): *δ* 12.35 (bs, 1H, COOH), 7.80 (s, 1H, ArH), 7.72 (d, *J* = 8.5 Hz, 1H, ArH), 7.26 (dd, *J* = 14.0 Hz, 2.5 Hz, 2H, ArH), 7.13 (d, *J* = 8.5 Hz, 1H, ArH), 7.01 (t, *J* = 8.0 Hz, 2H, ArH), 6.14 (s, 1H, CH), 5.34 (d, *J* = 2.0 Hz, 1H, CH), 5.21 (d, *J* = 2.0 Hz, 1H, CH); ^13^C NMR (DMSO, 400 MHz): *δ* 178.2, 144.8, 140.8, 138.1, 133.2, 132.0, 131.7, 128.9, 127.9, 125.4, 115.6, 84.7, 71.2; Anal. Calcd. for C_15_H_11_FO_3_: C, 69.76; H, 4.29. Found: C, 69.70; H, 4.24.

#### 2.1.5. Procedure for the Synthesis of 1-(4-Fluorophenyl)-1,3-dihydroisobenzofuran-5-carbohydrazide (10)

1-(4-Fluorophenyl)-1,3-dihydroisobenzofuran-5-carboxylic acid** 8** (5 g, 0.019 mol) is refluxed with ethanol (20 mL) and catalytic amount of Conc. H_2_SO_4_. The mixture is refluxed for 5 h. The progress of the reaction is monitored by TLC (toluene : ethyl acetate = 7.5 : 2.5). To stop the back reaction, excess of alcohol is evaporated off by rotavap. Residue is extracted with diethyl ether. Ether layer is washed with 5% sodium bicarbonate solution and thereafter evaporated to yield corresponding aromatic ester** 9**. Compound** 9** (4 g, 0.013 mol) is directly refluxed with 98% hydrazine hydrate (4 mL) in ethanol (4 mL) for 3 h. The progress of the reaction is monitored by TLC (toluene : ethyl acetate : diethylamine = 7.5 : 2.5 : 1). Reaction mixture is cooled and the solid formed is filtered and washed with ethanol to get 1-(4-fluorophenyl)-1,3-dihydroisobenzofuran-5-carbohydrazide** 10** as white solid with a yield of 3.6 g (94%), m.p. 238–240°C; IR (KBr pellet, cm^−1^): *ν* 3185 (NH), 1695 (C=O), 1658, 1477 (C=C), 1110 (hydrazine N–N), 1083 (COC); ^1^H NMR (DMSO, 400 MHz): *δ* 11.91 (s, 1H, NH), 11.76 (s, 2H, NH_2_), 8.48 (s, 1H, ArH), 7.93 (d, *J* = 8.5 Hz, 1H, ArH), 7.74 (dd, *J* = 14.0 Hz, 2.5 Hz, 2H, ArH), 7.42 (d, *J* = 8.5 Hz, 1H, ArH), 7.21 (t, *J* = 8.0 Hz, 2H, ArH), 6.27 (s, 1H, CH), 5.34 (d, *J* = 2.0 Hz, 1H, CH), 5.21 (d, *J* = 2.0 Hz, 1H, CH); ^13^C NMR (DMSO, 400 MHz): *δ* 161.0, 147.8, 139.7, 133.8, 129.5, 129.1, 127.5, 127.1, 122.6, 121.3, 125.0, 115.9, 84.5, 72.8; Anal. Calcd. for C_15_H_13_ FN_2_O_2_: C, 66.17; H, 4.81; N, 10.29. Found: C, 66.19; H, 4.77; N, 10.22.

#### 2.1.6. Representative Procedure for the Synthesis of 1-(4-Fluorophenyl)-1,3-dihydroisobenzofuran-5-acyl Thiosemicarbazides **11**(**a–h**)

Reflux an equimolar mixture of compound** 10** and 4-substituted phenyl isothiocyanate (**a–h**) in methanol (10 vol.) for 3 h. After cooling, the resulting solid is filtered and recrystallized from methanol to afford pure acyl thiosemicarbazides** 11**(**a–h**).

#### 2.1.7. Typical Procedure for the Synthesis of 4-Substituted Phenyl-5-[1-(4-fluorophenyl)-1,3-dihydroisobenzofuran-5-yl]-2H-1,2,4-triazole-3-thiones **12**(**a–h**)

Thiosemicarbazide** 11**(**a–h**) (1.0 mmol) is added portionwise to 5% NaOH solution (30 mL) and the reaction mixture is refluxed for 4 h. Cool and acidify with 6 N HCl to pH 2-3. The precipitated solid** 12**(**a–h**) is filtered, washed with water, and recrystallized from ethanol.

The same procedure is used in all the following cases.

*(1) Synthesis of 4-Phenyl-5-[1-(4-fluorophenyl)-1,3-dihydroisobenzofuran-5-yl]-2H-1,2,4-triazole-3-thione* ***12a***. From** 11a** (0.50 g, 1.23 mmol) we synthesized a white solid** 12a** (0.436 g, 91%) with m.p. 208–210°C; IR (KBr pellet, cm^−1^): *ν* 3402 (NH), 3085 (aromatic CH), 1604 (C=N), 1404 (C=C), 1234 (C=S), 1157 (COC), 1033 (CF); ^1^H NMR (DMSO, 400 MHz): *δ* 14.14 (s, 1H, NH), 7.50 (s, 1H, ArH), 7.48 (d, *J* = 8.5 Hz, 1H, ArH), 7.37 (dd, *J* = 14.0 Hz, 2.6 Hz, 2H, ArH), 7.34 (t, *J* = 8.0 Hz, 2H, ArH), 7.33 (d, *J* = 8.5 Hz, 1H, ArH), 7.18–7.0 (m, 5H, ArH), 6.15 (s, 1H, CH), 5.23 (d, *J* = 2.0 Hz, 1H, CH), 5.17 (d, *J* = 2.0 Hz, 1H, CH); ^13^C NMR (DMSO, 400 MHz): *δ* 169.0, 150.9, 144.4, 139.7, 134.9, 129.9, 129.8, 129.1, 128.9, 128.8, 128.2, 125.8, 122.6, 121.9, 115.8, 115.6, 84.3, 72.7; EI-MS (110 V)* m*/*z* (%): 390 (M^+^, 100), 360 (6.7), 267 (28), 165 (2.5), 123 (4.3); Anal. Calcd. for C_22_H_16_ FN_3_OS: C, 67.85; H, 4.14; N 10.79. Found: C, 67.82; H, 4.19; N 10.74.

*(2) Synthesis of 4-Benzyl-5-[1-(4-fluorophenyl)-1,3-dihydroisobenzofuran-5-yl]-2H-1,2,4-triazole-3-thione* ***12b***. From** 11b** (0.50 g, 1.19 mmol) we synthesized a white solid** 12b** (0.432 g, 90%) with m.p. 194–196°C; IR (KBr pellet, cm^−1^): *ν* 3448 (NH), 3085 (aromatic CH), 2923 (CH), 1654 (C=N), 1504 (C=C), 1280 (C=S), 1033 (CF); ^1^H NMR (DMSO, 400 MHz): *δ* 14.11 (s, 1H, NH), 7.53 (s, 1H, ArH), 7.37 (d, *J* = 8.5 Hz, 1H, ArH), 7.34 (dd, *J* = 14.0 Hz, 2.5 Hz, 2H, ArH), 7.24 (t, *J* = 8.0 Hz, 2H, ArH), 7.19 (d, *J* = 8.5 Hz, 1H, ArH), 7.0–7.11 (m, 5H, ArH), 6.21 (s, 1H, CH), 5.28 (d, *J* = 2.0 Hz, 1H, CH), 5.44 (d, *J* = 2.0 Hz, 1H, CH), 2.5 (s, 2H, CH_2_); ^13^C NMR (DMSO, 400 MHz): *δ* 168.5, 151.7, 144.8, 140.0, 138.8, 136.2, 129.9, 129.1, 129.0, 128.4, 127.9, 126.0, 123.0, 122.1, 115.9, 115.6, 85.4, 72.7, 47.1; Anal. Calcd. for C_23_H_18_ FN_3_OS: C, 68.47; H, 4.50; N, 10.41. Found: C, 68.42; H, 4.57; N, 10.43.

*(3) Synthesis of 4-(2-Phenylethyl)-5-[1-(4-fluorophenyl)-1,3-dihydroisobenzofuran-5-yl]-2H-1,2,4-triazole-3-thione* ***12c***. From** 11c** (0.50 g, 1.15 mmol) we synthesized a white solid** 12c** (0.413 g, 86%) with m.p. 212–214°C; IR (KBr pellet, cm^−1^): *ν* 3271 (NH), 3155 (aromatic CH), 1666 (C=N), 1542 (C=C), 1234 (C=S), 1164 (COC), 1018 (CF); ^1^H NMR (DMSO, 400 MHz): *δ* 14.13 (s, 1H, NH), 7.50 (s, 1H, ArH), 7.36 (d, *J* = 8.5 Hz, 1H, ArH), 7.32 (dd, *J* = 14.0 Hz, 2.5 Hz, 2H, ArH), 7.29 (t, *J* = 8.0 Hz, 2H, ArH), 7.15 (d, *J* = 8.5 Hz, 1H, ArH), 7.0–7.08 (m, 5H, ArH), 6.20 (s, 1H, CH), 5.24 (d, *J* = 2.0 Hz, 1H, CH), 5.47 (d, *J* = 2.0 Hz, 1H, CH), 3.6 (t, *J* = 7.0 Hz, 2H, CH_2_), 2.51 (t, *J* = 7.0 Hz, 2H, CH_2_); ^13^C NMR (DMSO, 400 MHz): *δ* 169.1, 150.6, 144.2, 140.6, 135.1, 129.5, 129.1, 128.8, 128.2, 127.9, 126.3, 125.4, 122.9, 121.1, 115.6, 115.1, 85.0, 72.7, 48.4, 33.2; Anal. Calcd. for C_24_H_20_ FN_3_OS: C, 69.04; H, 4.83; N, 10.06. Found: C, 69.06; H, 4.88; N, 10.04.

*(4) Synthesis of 4-(4-Chlorophenyl)-5-[1-(4-fluorophenyl)-1,3-dihydroisobenzofuran-5-yl]-2H-1,2,4-triazole-3-thione* ***12d***. From** 11d** (0.50 g, 1.13 mmol) we synthesized a white solid** 12d** (0.387 g, 79%) with m.p. 180–182°C; IR (KBr pellet, cm^−1^): *ν* 3440 (NH), 3093 (aromatic CH), 1612 (C=N), 1488 (C=C), 1226 (C=S), 1103 (COC), 1033 (CF), 825 (CCl); ^1^H NMR (DMSO, 400 MHz): *δ* 14.07 (s, 1H, NH), 7.92 (s, 1H, ArH), 7.88 (d, *J* = 8.5 Hz, 1H, ArH), 7.70 (d, *J* = 7.0 Hz, 2H, ArH), 7.64 (d, *J* = 7.0 Hz, 2H, ArH), 7.45 (dd, *J* = 14.0 Hz, 2.5 Hz, 2H, ArH), 7.31 (t, *J* = 8.0 Hz, 2H, ArH), 7.25 (d, *J* = 8.5 Hz, 1H, ArH), 6.21 (s, 1H, CH), 5.16 (d, *J* = 2.0 Hz, 1H, CH), 5.10 (d, *J* = 2.0 Hz, 1H, CH); ^13^C NMR (DMSO, 400 MHz): *δ* 169.0, 152.6, 147.5, 144.5, 139.8, 138.7, 134.5, 131.1, 129.8, 128.9, 128.8, 128.3, 125.7, 122.7, 115.8, 155.6, 84.3, 72.7; Anal. Calcd. for C_22_H_15_ ClFN_3_OS: C, 62.34; H, 3.57; N, 9.91. Found: C, 69.32; H, 3.54; N, 9.96.

*(5) Synthesis of 4-(4-Nitrophenyl)-5-[1-(4-fluorophenyl)-1,3-dihydroisobenzofuran-5-yl]-2H-1,2,4-triazole-3-thione* ***12e***. From** 11e** (0.50 g, 1.14 mmol) we synthesized a white solid** 12e** (0.416 g, 83%) with m.p. 174–176°C; IR (KBr pellet, cm^−1^): *ν* 3224 (NH), 3091 (aromatic CH), 1612 (C=N), 1481 (C=C), 1315 (NO_2_), 1244 (C=S), 1103 (COC); ^1^H NMR (DMSO, 400 MHz): *δ* 14.19 (s, 1H, NH), 8.20 (d, *J* = 8.0 Hz, 2H, ArH), 7.94 (s, 1H, ArH), 7.83 (d, *J* = 8.5 Hz, 1H, ArH), 7.42 (d, *J* = 8.0 Hz, 2H, ArH), 7.37 (dd, *J* = 14.0 Hz, 2.5 Hz, 2H, ArH), 7.30 (t, *J* = 8.0 Hz, 2H, ArH), 7.24 (d, *J* = 8.5 Hz, 1H, ArH), 6.87 (s, 1H, CH), 5.21 (d, *J* = 2.0 Hz, 1H, CH), 5.18 (d, *J* = 2.0 Hz, 1H, CH); ^13^C NMR (DMSO, 400 MHz): *δ* 168.8, 153.6, 150.6, 143.8, 137.9, 134.5, 129.9, 129.5, 129.1, 128.6, 127.3, 125.8, 124.4, 122.5, 117.0, 115.2, 84.2, 72.1; Anal. Calcd. for C_22_H_15_ FN_4_O_3_S: C, 60.82; H, 3.48; N, 12.90. Found: C, 60.87; H, 3.43; N, 12.96.

*(6) Synthesis of 4-(4-Methylphenyl)-5-[1-(4-fluorophenyl)-1,3-dihydroisobenzofuran-5-yl]-2H-1,2,4-triazole-3-thione* ***12f***. From** 11f** (0.50 g, 1.19 mmol) we synthesized a white solid** 12f** (0.422 g, 88%) with m.p. 152–154°C; IR (KBr pellet, cm^−1^): *ν* 3162 (NH), 3044 (aromatic CH), 2948 (CH), 1612 (C=N), 1472 (C=C), 1226 (C=S), 1146 (COC), 1012 (CF); ^1^H NMR (DMSO, 400 MHz): *δ* 14.07 (s, 1H, NH), 7.92 (s, 1H, ArH), 7.88 (d, *J* = 8.5 Hz, 1H, ArH), 7.64 (d, *J* = 7.0 Hz, 2H, ArH), 7.58 (d, *J* = 7.0 Hz, 2H, ArH), 7.40 (dd, *J* = 14.0 Hz, 2.6 Hz, 2H, ArH), 7.33 (t, *J* = 8.0 Hz, 2H, ArH), 7.26 (d, *J* = 8.5 Hz, 1H, ArH), 6.12 (s, 1H, CH), 5.20 (d, *J* = 2.0 Hz, 1H, CH), 5.17 (d, *J* = 2.0 Hz, 1H, CH), 2.33 (s, 3H, CH_3_); ^13^C NMR (DMSO, 400 MHz): *δ* 169.0, 150.5, 144.7, 143.6, 133.0, 130.3, 129.8, 129.7, 129.1, 128.9, 128.3, 126.8, 125.7, 122.6, 119.5, 115.8, 83.7, 72.5, 21.4; Anal. Calcd. for C_23_H_18_ FN_3_OS: C, 68.47; H, 4.50; N, 10.41. Found: C, 68.42; H, 4.56; N, 10.47.

*(7) Synthesis of 4-(4-Methoxyphenyl)-5-[1-(4-fluorophenyl)-1,3-dihydroisobenzofuran-5-yl]-2H-1,2,4-triazole-3-thione* ***12g***. From** 11g** (0.50 g, 1.14 mmol) we synthesized a white solid** 12g** (0.403 g, 84%) with m.p. 174–176°C; IR (KBr pellet, cm^−1^): *ν* 3210 (NH), 3093 (aromatic CH), 1646 (C=N), 1428 (C=C), 1305 (CO), 1278 (C=S), 1140 (COC), 1005 (CF); ^1^H NMR (DMSO, 400 MHz): *δ* 14.10 (s, 1H, NH), 7.99 (s, 1H, ArH), 7.85 (d, *J* = 8.5 Hz, 1H, ArH), 7.44 (dd, *J* = 14.0 Hz, 2.6 Hz, 2H, ArH), 7.32 (t, *J* = 8.0 Hz, 2H, ArH), 7.24 (d, *J* = 8.5 Hz, 1H, ArH), 7.11 (d, *J* = 7.5 Hz, 2H, ArH), 6.79 (d, *J* = 7.5 Hz, 2H, ArH), 6.44 (s, 1H, CH), 5.29 (d, *J* = 2.0 Hz, 1H, CH), 5.01 (d, *J* = 2.0 Hz, 1H, CH), 3.11 (s, 3H, OCH_3_); ^13^C NMR (DMSO, 400 MHz): *δ* 168.4, 150.4, 150.1, 144.7, 140.0, 134.1, 131.1, 129.5, 129.1, 128.9, 128.2, 124.3, 122.0, 117.1, 117.4, 115.4, 84.1, 72.8, 53.6; Anal. Calcd. for C_23_H_18_ FN_3_O_2_S: C, 65.86; H, 4.33; N, 10.02. Found: C, 65.82; H, 4.39; N, 10.07.

*(8) Synthesis of 4-(4-Bromophenyl)-5-[1-(4-fluorophenyl)-1,3-dihydroisobenzofuran-5-yl]-2H-1,2,4-triazole-3-thione* ***12h***. From** 11h** (0.50 g, 1.05 mmol) we synthesized a white solid** 12h** (0.398 g, 81%) with m.p. 206–208°C; IR (KBr pellet, cm^−1^): *ν* 3241 (NH), 3077 (aromatic CH), 1672 (C=N), 1488 (C=C), 1237 (C=S), 1142 (COC), 1075 (CF), 524 (CBr); ^1^H NMR (DMSO, 400 MHz): *δ* 14.03 (s, 1H, NH), 7.90 (s, 1H, ArH), 7.85 (d, *J* = 8.5 Hz, 1H, ArH), 7.66 (d, *J* = 7.0 Hz, 2H, ArH), 7.52 (d, *J* = 7.0 Hz, 2H, ArH), 7.44 (dd, *J* = 14.0 Hz, 2.5 Hz, 2H, ArH), 7.32 (t, *J* = 8.0 Hz, 2H, ArH), 7.24 (d, *J* = 8.5 Hz, 1H, ArH), 6.19 (s, 1H, CH), 5.18 (d, *J* = 2.0 Hz, 1H, CH), 5.12 (d, *J* = 2.0 Hz, 1H, CH); ^13^C NMR (DMSO, 400 MHz): *δ* 168.1, 150.6, 146.2, 145.1, 134.6, 133.2, 129.9, 129.5, 129.0, 128.8, 127.1, 125.7, 122.4, 118.4, 115.2, 113.6, 84.3, 72.6; Anal. Calcd. for C_22_H_15_ Br FN_3_OS: C, 56.42; H, 3.23; N, 8.97. Found: C, 56.44; H, 3.26; N, 8.91.

### 2.2. Biology

As per NCCLS document M62-A7 Protocols, the preliminary antimicrobial activity is performed using the disc diffusion method [22]. Four bacterial and four fungal strains are chosen for the study. They are* Bacillus subtilis* (NCIM 2063),* Staphylococcus aureus* (NCIM 2079),* Escherichia coli* (NCIM 2574),* Pseudomonas aeruginosa* (NCIM 2036),* Candida albicans* (NCIM 3471),* Saccharomyces cerevisiae* (NCIM 3559),* Aspergillus flavus* (NCIM 1316), and* Aspergillus niger* (NCIM 545). The bacterial strains are maintained on Muller-Hinton (MH) agar medium and fungi are sustained on Sabouraud Dextrose (SD) agar medium.

The bacterial inoculum is prepared by suspending in 9 mL of sterile water for colonies from 24 h culture on MH agar medium. For the fungi, the inoculum is prepared with spores derived from 48 h–96 h culture on SD agar medium.

To standardize the inoculum density for a susceptibility test, a BaSO_4_ turbidity standard, equivalent to a 0.5 McFarland is used. The turbidity of the actively growing microbial culture is adjusted with sterile saline to attain turbidity optically comparable to that of the 0.5 McFarland standards. Within 15 minutes after adjusting the turbidity of the inoculum suspension, the petri plates are inoculated with a standardized suspension of the tested microorganisms. The predetermined series of antimicrobial discs is allotted onto the surface of the inoculated agar plate. Whatman filter paper number 1 is used to prepare discs approximately 6 mm in diameter, which are sterilized in a hot air oven before placing in petri plates. These paper discs are placed in a circular pattern in each inoculated plate and then injected with the specific amount of tested compound using micropipettes.

All the newly synthesized mercaptotriazoles are dissolved in dimethyl sulfoxide (DMSO) to prepare chemicals of stock solution of 10 mg/1 mL for antibacterial study and 25 mg/1 mL for antifungal study. Gentamicin and fluconazole are used as standard antibacterial and antifungal drugs, respectively. Dimethyl sulfoxide is used as solvent control. The plates are incubated and the inhibitory activity is measured (in mm) as the diameter of the observed inhibition zones. The tests are repeated to confirm the findings and the average of the readings is taken into account. The results of study are tabulated in Tables 1 and 2.

Following M62-A7 Protocols, broth dilution test by doubling dilution of the antibiotics is carried out to examine the minimum inhibitory concentration of the most promising compound** 12d** against the tested microbes. The antibacterial and antifungal assays are performed in Mueller-Hinton broth and Sabouraud Dextrose broth, respectively. MIC for microbes is to be observed between 250 *μ*g/1 mL and 7.81 *μ*g/1 mL. Gentamicin (1 mg/2 mL) and fluconazole (1 mg/2 mL) are used as standard antibacterial and antifungal drugs, respectively. DMSO is the solvent control.

## 3. Results and Discussion

**Scheme 1:**
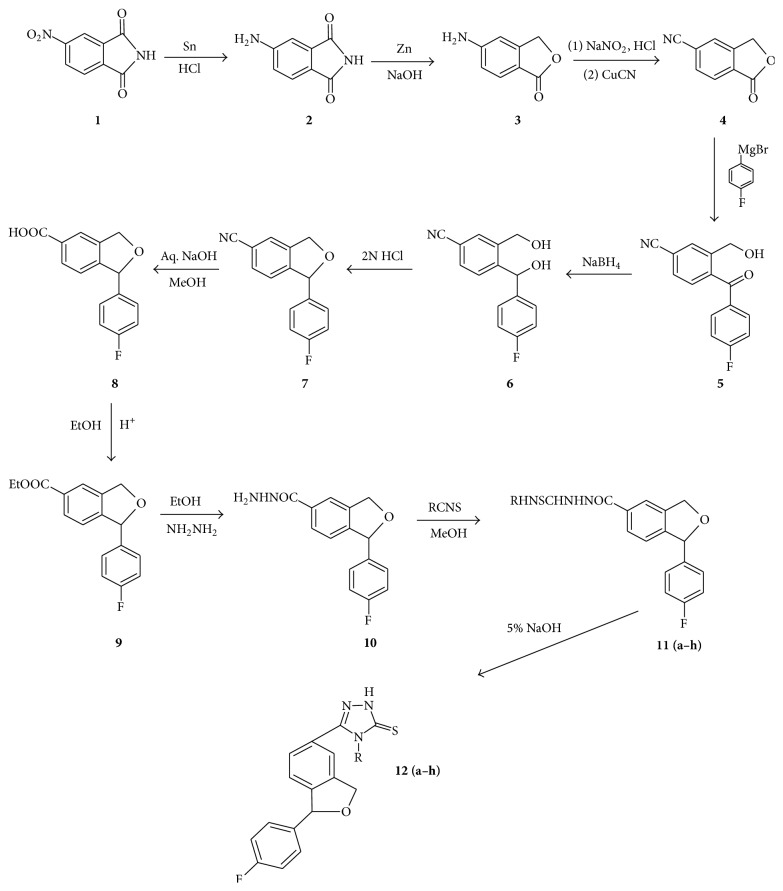
*R* = (a) phenyl, (b) benzyl, (c) phenethyl, (d) 4-chlorophenyl, (e) 4-nitrophenyl, (f) 4-methylphenyl, (g) 4-methoxyphenyl, and (h) 4-bromophenyl.

4-Nitrophthalimide** 1** is reduced to 4-aminophthalimide** 2** using SnCl_2_/HCl which is converted to 5-amino-3H-isobenzofuran-1-one** 3** by employing Zn/NaOH. Compound** 3** is altered to 1-oxo-1,3-dihydro-isobenzofuran-5-carbonitrile** 4** by Sandmeyer reaction. Compound** 4**, commonly called 5-cyanophthalide, is subjected to Grignard reaction with 4-fluorophenyl magnesium bromide in tetrahydrofuran and the resulting product is treated with sodium borohydride to obtain the diol** 6**, which is cyclized with 2N HCl to afford 1-(4-fluorophenyl)-1,3-dihydroisobenzofuran-5-carbonitrile** 7**. Compound** 7** is hydrolysed in aq. methanolic NaOH to provide 1-(4-fluorophenyl)-1,3-dihydroisobenzofuran-5-carboxylic acid** 8** in good yield. This acid is esterified and the ester** 9** is converted to 1-(4-fluorophenyl)-1,3-dihydroisobenzofuran-5-carbohydrazide** 10**. The synthesized acid hydrazide is treated with different substituted phenyl isothiocyanates (**a–h**) to yield 1-(4-fluorophenyl)-1,3-dihydroisobenzofuran-5-acyl thiosemicarbazides** 11**(**a–h**). Cyclodehydration of the thiosemicarbazides** 11**(**a–h**) in basic medium (5% NaOH) leads to the formation of 4-substituted phenyl-5-[1-(4-fluorophenyl)-1,3-dihydroisobenzofuran-5-yl]-2H-1,2,4-triazole-3-thiones** 12**(**a–h**) (Scheme 1).

Purity of the compounds is analyzed by TLC, LCMS, and elemental analyses. ^1^H NMR, ^13^C NMR, and IR spectra have backed up the characterization. The IR spectra of the mercaptotriazoles** 12**(**a–h**) mark the absence of carbonyl absorption around 1750 cm^−1^. The existence of triazoles in thione form is indicated by the absence of SH stretching in the characteristic region of 2540 cm^−1^. The ^1^H NMR spectrum of every compound unveils the deshielding of NH of the triazole ring as anticipated in the range *δ* 14.00–14.50. The signal for NH appearing downfield than *δ* 12.0 authenticates the existence of the ring in thione form. The characteristic fluorocoupling is observed for the aromatic protons of the phenyl ring attached to the 1st position of isobenzofuran moiety. In the ^13^C NMR spectra of triazol-3-thiones, values close to *δ* 168 and *δ* 150 are the characteristic signals for C-3 and C-5 carbons, respectively. Disappearance of signal around *δ* 161–163 for carbonyl carbons indicates the formation of target compounds** 12**(**a–h**). The molecular ion peak in the mass spectrum of C_22_H_16_FN_3_OS (**12a**) is found to be at* m*/*z* 390.09. This is in agreement with its calculated formula weight 389.44. This confirms the formation of the product. The base peak is observed at* m*/*z* 267.07 for C_15_H_11_N_3_S fragment.

Many research articles on heterocycles have explicated the synthesis of 2-amino-5-substituted-1,3,4-thiadiazole derivatives from substituted acyl thiosemicarbazides. This reaction runs parallel to the preparation of 3-mercapto-1,2,4-triazoles from the same starting material. Here, we have followed the classical methods of cyclization with cold concentrated H_2_SO_4_. Moreover, the use of novel reagents like saccharin and* p*-toluene sulfonic acid has also failed to afford the required products. The spectra have disclosed the decomposition of substituted thiosemicarbazides. Therefore, this plan of synthesis is discontinued. Similarly, spectral characterization reveals that 2-substituted acyl thiosemicarbazides have failed to form the required mercaptotriazoles. Greater steric hindrance of the bulky isobenzofuran substituents may be the rationalization for both failures.

Disc diffusion method is followed to observe the antimicrobial action. The results of the preliminary testing of the antimicrobial activity of the novel compounds are tabulated in Tables 1 and 2. Varying grades of inhibition against the growth of the tested microbes by the synthesized compounds can be noticed. Broadly, the inhibitory activity against the Gram-positive bacteria is higher than against the Gram-negative bacteria. Compound** 12d** shows significant inhibition against all the tested microorganisms owing to the presence of chloro group on the benzene ring attached to the 4th position of triazole ring. The better activity of** 12e**,** 12g**, and** 12h** can be attributed to the presence of nitro, methoxy, and bromo groups, respectively, at para positions of benzene ring. Compound** 12a**, which has unsubstituted benzene at 4th position of triazole ring, also shows better activity than the triazoles with benzyl and phenylethyl groups. The fluoro substituent attached to the* para* position of benzene ring, which is connected to the 1st position of the isobenzofuran core, appears to be the reason behind the antimicrobial activities displayed by the target compounds. Minimum inhibitory concentration (MIC) is inspected for** 12d** by broth dilution approach. For Gram-positive bacteria, the values are less than 62.5, but for Gram-negative bacteria the results are 125 ≤ MIC > 62.5. For all the fungal strains, the values are less than 62.5.

## 4. Conclusion

The present work is a multistep synthesis of a novel series of 4-substituted phenyl-5-[1-(4-fluorophenyl)-1,3-dihydroisobenzofuran-5-yl]-2H-1,2,4-triazole-3-thiones. Structural confirmation is done with analytical techniques and antimicrobial action is performed by disc diffusion method. Based on structural activity relationship study, compounds** 12a**,** 12d**,** 12e**,** 12g**, and** 12h** have shown good inhibition against microbes.

## Figures and Tables

**Table 1:** Inhibitory zone (diameter) (in mm) of synthesized compounds against tested bacterial strains by disc diffusion method.

Compound	*Bacillus subtilis *	*Staphylococcus aureus *	*Escherichia coli *	*Pseudomonas aeruginosa *
**12a**	23	21	14	10
**12b**	18	16	12	08
**12c**	17	14	11	06
**12d**	30	25	19	14
**12e**	24	21	14	11
**12f**	18	20	12	10
**12g**	25	19	14	10
**12h**	24	22	15	12
Gentamicin	27	26	22	18

Synthesized compound taken is 10 *μ*L of 10 mg/1 mL and gentamicin (10 *μ*g per disc) is the positive reference standard antibiotic disc.

**Table 2:** Inhibitory zone (diameter) (in mm) of synthesized compounds against tested fungal strains by disc diffusion method.

Compound	*Candida albicans *	*Saccharomyces cerevisiae *	*Aspergillus flavus *	*Aspergillus niger *
**12a**	22	16	07	09
**12b**	16	09	05	03
**12c**	13	06	04	03
**12d**	28	19	11	13
**12e**	24	15	14	10
**12f**	16	12	05	04
**12g**	22	16	08	10
**12h**	24	16	09	11
Fluconazole	24	19	10	08

Synthesized compound taken is 10 *μ*L of 25 mg/1 mL and fluconazole (25 *μ*g per disc) is the positive reference standard antifungal disc.
